# Geographically distinct patterns of reproductive isolation and hybridization in two sympatric species of the *Jaera albifrons* complex (marine isopods)

**DOI:** 10.1002/ece3.3106

**Published:** 2017-06-09

**Authors:** Ambre Ribardière, Claire Daguin‐Thiébaut, Céline Houbin, Jérôme Coudret, Caroline Broudin, Olivier Timsit, Thomas Broquet

**Affiliations:** ^1^ Centre National de la Recherche Scientifique Team Diversity and Connectivity of Coastal Marine Landscapes Roscoff France; ^2^ Sorbonne Universités UPMC Univ Paris 06 Roscoff France; ^3^ Centre National de la Recherche Scientifique Roscoff France; ^4^ Groupe d'Etude des Milieux Estuariens et Littoraux de Normandie Centre Régional d'Etudes Côtières Luc‐sur‐Mer France

**Keywords:** crustaceans, genetic incompatibilities, introgression, isolating barriers, Mosaic hybrid zone, sexual isolation

## Abstract

Sympatric species that in some populations hybridize and in other populations remain reproductively isolated open interesting research possibilities for the study of hybridization and speciation. Here, we test for such a situation in two littoral isopods (*Jaera albifrons* and *J. praehirsuta*) that occur in mixed populations and where past morphological descriptions suggested that the two species are generally reproductively isolated except in rare populations where hybridization may be happening. Using field surveys and microsatellite genetic structure analyses in two regions from France (Normandy and Brittany), we confirmed that introgressive hybridization occurs in a subset of mixed *J. albifrons*/*J. praehirsuta* populations (region Normandy) where the two species are found in the same habitat (pebbles on the shore). Moreover, we found that introgression in these populations is differential, 21 of 23 microsatellite markers showing little genetic divergence between species (hierarchical analysis of molecular variance *F*_CT_ = 0.017) while the remaining two loci were strongly differentiated (*F*_CT_ = 0.428). By contrast, *J. albifrons* and *J. praehirsuta* in mixed populations from region Brittany occupied distinct habitats (pebbles and seaweeds, respectively) with little overlap and showed stronger genetic divergence (*F*_CT_ = 0.132). In hybridizing populations, the majority of individuals show morphological traits that are characteristic of one or the other species. This raises the question of the forces that act to maintain this polymorphism, noting that hybridizing populations seem to be geographically isolated from potential source parental populations and show no detectable habitat divergence between species.

## 1 INTRODUCTION

Natural hybridization events inform our understanding of isolating barriers between species, the conditions of species coexistence despite hybridization, and the mechanisms of speciation. The archetypal hybrid zone structure is a region of contact between two otherwise allopatric species. In such hybrid zones, flanked on one side by populations of one species and on the other side by the other species, the dynamics of the system is most often driven by a balance between immigration from pure parental populations and selection against hybrids (the tension zone model, Barton & Hewitt, 1985). In such systems, individuals freely hybridize in the contact zone and hybrids have reduced fitness due to the segregation of genetic incompatibilities. Studies of naturally hybridizing populations have also increasingly highlighted the role of other isolating barriers, including environmentally induced selection (Arnold, 1997; Endler, 1977; Moore, 1977) and sexual isolation (Poelstra et al., 2014; Seehausen, vanAlphen, & Witte, 1997).

Hybridizing populations vary not only in the nature of isolating mechanisms that are involved but also in geographic structure. Hybrid zones are typically characterized by a clinal structure (gradients of allelic frequencies between pure parental populations). More complex spatial structures are found when the environment induces differential selection on hybridizing species and the distribution of habitats is discrete (e.g., islands, lakes, host plants) or otherwise heterogeneous, leading to patchy hybrid zones (mosaic hybrid zones, Harrison & Rand, 1989; and other types of replicated hybridizing populations, reviewed, e.g., in Harrison & Larson, 2016). Variable degrees of patchiness can also be induced by colonization history or population stochasticity (Gompert, Lucas, Fordyce, Forister, & Nice, 2010). Whatever causes patchiness, patchy systems allow us to compare multiple, potentially independent contact zones (Bierne et al., 2003; Butlin et al., 2014; McKinnon & Rundle, 2002). Such comparisons are also possible in hybrid zones that have a simpler spatial structure but that can be sampled along replicated transects (e.g., Teeter et al., 2010), and, notably, in experimental populations (Pritchard & Edmands, 2013). These comparative analyses may increase our understanding of isolation mechanisms, their associated genomic architecture, and, promisingly, speciation (Harrison & Larson, 2016; Westram, Panova, Galindo, & Butlin, 2016).

Particularly intriguing are the situations where one can compare populations composed by a mixture of individuals of two species that in some instances hybridize and in other instances remain strongly reproductively isolated. That is, sympatric or mixed populations that may or may not be reproductively isolated; hereafter, we will use the term “mixed populations,” defined as populations where individuals of two species are close enough so that they can meet and interact frequently. An illustrative case in point is the lake Victoria cichlid “speciation transect” (Seehausen, 2009) where mixed populations of *Pundamilia pundamilia* and *P. nyererei* show more or less hybridization depending on variations in premating behavioral mechanisms themselves linked with variations in habitat (water clarity). Fish studies have provided a few other related examples where a pair of species shows contrasted levels of reproductive isolation when in sympatry (benthic and limnetic three‐spined stricklebacks, Taylor et al., 2006; swordtail fish, Culumber et al., 2011; and lake whitefish, Gagnaire, Pavey, Normandeau, & Bernatchez, 2013; river and blueback herring, Hasselman et al., 2014; and river and brook lampreys, Rougemont et al., 2015). Comparing sympatric nonhybridizing/hybridizing populations provides power to interpret admixture patterns (e.g., shared ancestral polymorphism vs. current gene flow) or assess whether differential introgression patterns are due to heterogeneous recombination, selection, or gene flow (Gagnaire et al., 2013; Powell et al., 2013; Rougemont et al., 2016).

Here we focus on the *Jaera albifrons* group, a complex of five marine isopod species that live on the shores of the temperate and cold waters of the North‐Atlantic Ocean. It includes *J. albifrons*,* J. praehirsuta*,* J. ischiosetosa*,* J. forsmani*, and *J. posthirsuta* (Bocquet, 1953, 1972; Naylor & Haahtela, 1966). Note that *Jaera albifrons* designates one of the five species of the *Jaera albifrons* group (the distinction will be noted using the words “complex” or “group” throughout). All five species occupy a narrow but geographically extended belt in the intertidal zone and they have largely overlapping distribution ranges. In short, individuals from one species frequently coexist with individuals from at least one other species throughout their distribution range, and mixed populations are the rule rather than the exception. In this context, the five species of the *Jaera albifrons* group were shown to be reproductively isolated by at least three types of barriers: (1) ecological isolation (variations in local habitat preferences along the seashore), (2) sexual isolation (differences in male secondary sexual traits used in tactile courtship, and strong female‐driven mate choice), and (3) genetic incompatibilities (reviewed in Solignac, 1978, 1981; Mifsud, 2011). The reproductive isolation resulting from the combination of these pre‐ and postzygotic barriers is thought to be very strong in nature.

However, intermediate male sexual traits have been reported in a few populations, suggesting that hybridization may happen in some rare places (Solignac, 1978). One such potentially hybridizing population has been intensively studied by Charles Bocquet and Michel Solignac between 1965 and 1970. They described a *Jaera albifrons*/*J. praehirsuta* mixed population located in Luc‐sur‐Mer, Normandy (France) where the analysis of male secondary sexual traits and experimental crosses led them to conclude that this population contained an exceptional proportion of hybrids (15%–32% depending on sampling event and classification thresholds, Bocquet & Solignac, 1969; Solignac, 1969a,b, 1978). Based on morphological descriptions for a large number of individuals sampled or raised in the laboratory from this population (nearly 2000 ind., Solignac, 1978) and comparison with experimental crosses (Bocquet & Solignac, 1969), their conclusion on hybridization between species seems very strong. This past work convincingly suggests that hybridization was occurring in at least one particular site in 1965–1970 while all other known *J. albifrons*/*J. praehirsuta* sympatric populations were reproductively isolated. As discussed above, such a situation seems interesting for the study of isolating barriers and speciation.

With this study, our objectives were (1) to test for hybridization between *J. albifrons* and *J. praehirsuta* using genetic tools, (2) to investigate the geographic structure of hybridizing populations and the nature of the isolating mechanisms, and (3) to compare genetic patterns within hybridizing vs. nonhybridizing mixed populations. For this purpose, we searched for mixed populations and morphologically intermediate individuals as described in Normandy ca. 50 years ago, analyzed the genetic structure of local populations using a panel of 23 microsatellite loci, and compared it with mixed populations from another French region (Brittany) where the two species had been described as reproductively isolated.

## 2 MATERIALS AND METHODS

### 2.1 Model species

**Figure 1:**
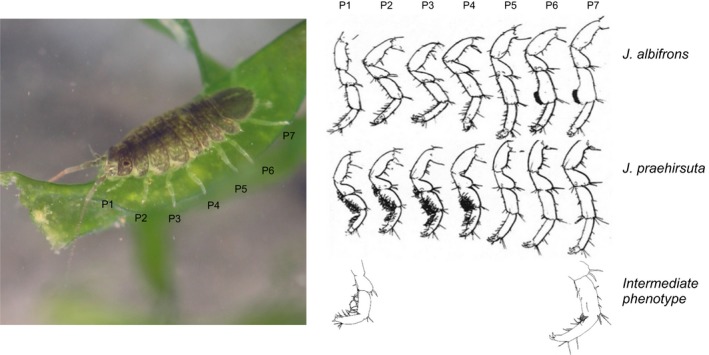
Morphological differentiation at peraeopods (numbered P1‐P7) between males *Jaera albifrons* (carpus of peraeopods P6 and P7 extended as a lobe with a number of straight setae) and *J. praehirsuta* (peraeopods P1‐4 with many curved setae) as found in region Brittany (western France, drawings reproduced from Solignac, 1981 with authorization). A few individuals with secondary sexual traits intermediate to *J. albifrons* and *J. praehirsuta* were found in region Normandy. The length of the individual (a female) represented on the picture is 4 mm. Photography credit to Guillaume Evanno & Thomas Broquet

Members of the *Jaera albifrons* complex are small marine crustaceans (2–5 mm total adult length, Figure 1). Of the five species comprising the *Jaera albifrons* complex, two are restricted to the temperate waters of the North‐American east coast (*J. posthirsuta*) or the European coast (*J. forsmani*), while the three others are more widely distributed on both sides of the Northern Atlantic (Bocquet, 1972). These five species are found in abundance in the intertidal zone, where they can show local habitat preferences involving variations in micro‐habitat (under rocks or on seaweeds), level on the intertidal zone, and salinity (Jones, 1972; Naylor & Haahtela, 1966). However, these habitat preferences vary widely, meaning that ecological isolation is also very variable (Solignac, 1981; Veuille, 1976).

The identification of species within the *Jaera albifrons* complex is based on male secondary sexual traits (Figure 1). Mating is preceded by a courtship behavior whereby males mount females in a head‐to‐tail position and used different parts of their peraeopods to brush or press the female's back. Males of the five species differ in the distribution of setae and spines borne by the peraeopods used to court females (Jones & Fordy, 1971; Solignac, 1978), and a female's acceptance or rejection is a major driver of reproductive isolation between species.

### 2.2 Species survey and sampling

**Figure 2:**
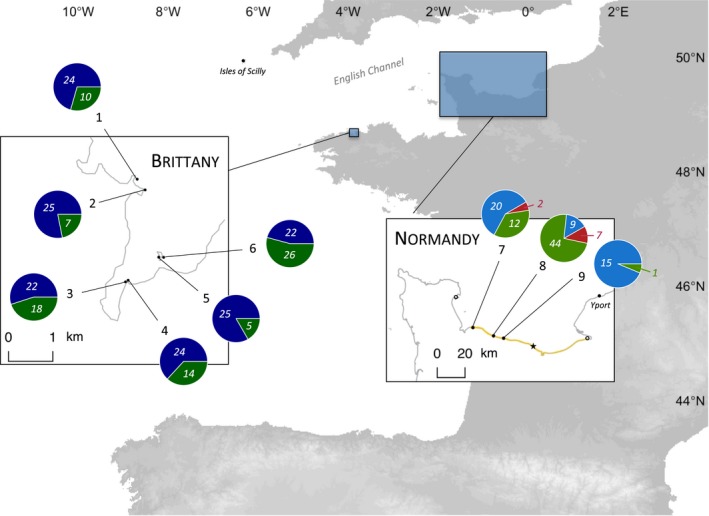
Sampling sites in two regions of Western France (“Brittany” and “Normandy”). Pie charts give the number of males showing secondary sexual traits typical of *Jaera albifrons* (in blue), *Jaera praehirsuta* (in green), and intermediate phenotypes (in red) sampled at each location. Note that these numbers reflect the relative proportions of each species at each site in Normandy, where the two species occupy the same microhabitat and cannot be distinguished in the field. By contrast, in Brittany, pie charts are representative of sample sizes but not necessary of the relative density of each species (because there the two species occupy two different habitats with little overlap, see text). Mixed populations from Normandy were found following an intensive survey (geographic extent shown in yellow, details in text), revealing that the hybridizing population originally studied by Michel Solignac in 1965–1970 (Luc‐sur‐Mer, indicated by a star) is now extinct. The nearest nonmixed populations that we could find comprised only *Jaera albifrons* (empty circles). Additional mixed populations with some individuals showing an intermediate phenotype were further found in Yport and the Isles of Scilly (black dots, see text)

We sampled *J. albifrons* and *J. praehirsuta* in two regions. First we focused on the area where Michel Solignac had described hybridization between these two species in 1965 and 1970 (Solignac, 1978). For that, we surveyed strictly all potential habitats on a 25‐km portion of the coast around this original site, finding the population studied by M. Solignac to be extinct (Luc‐sur‐Mer, Figure 2), possibly due to the regular removal of pebbles from the beach for touristic activities. We extended this survey 35 km East and 35 km West by visiting a large number of (but not strictly all) potential habitats, where we found three sites with a mixture of *J. albifrons* and *J. praehirsuta* (sites 7–9, see 3, Table 1, and Figure 2). This gave us a 95‐km continuous portion of the coastline where we have a precise, although not strictly exhaustive, view of the distribution of species (from Grandcamp‐Maisy to Honfleur, Second World War landing beaches, highlighted in yellow in Figure 2).

**Table 1:** *Jaera albifrons and J. praehirsuta* sampling locations, sample sizes, and genetic diversity (observed and expected heterozygosity *H*_o_ and *H*_e_, and *F*_IS_). Individuals with an intermediate phenotype (*n* = 9) from sites 7 and 8 are not included. The statistical significance of *F*_IS_ is indicated (*: *p*‐value < .05)

Sampled site	Coordinates	*J. albifrons*				*J. praehirsuta*			
		*N*	*H*_o_	*H*_*e*_	*F*_IS_	*N*	*H*_o_	*H*_e_	*F*_IS_
Brittany									
1	48°40′27.6″N, 3°57′11.3″W	24	0.48	0.55	0.137*	10	0.53	0.61	0.145
2	48°40′20.9″N, 3°57′00.3″W	25	0.47	0.57	0.188*	7	0.55	0.64	0.142
3	48°39′10.8″N, 3°57′03.0″W	22	0.51	0.54	0.070	18	0.54	0.62	0.135*
4	48°39′12.3″N, 3°57′00.4″W	24	0.5	0.57	0.131*	14	0.52	0.62	0.168*
5	48°39′34.1″N, 3°56′25.7″W	25	0.48	0.55	0.115	5	0.63	0.69	0.097
6	48°39′33.5″N, 3°56′31.2″W	22	0.45	0.52	0.132*	26	0.5	0.58	0.153*
Normandy									
7	49°23′30.4″N, 1°02′09.6″W	20	0.49	0.52	0.059	12	0.45	0.53	0.163*
8	49°21′15.7″N, 0°47′54.2″W	9	0.42	0.48	0.141	44	0.52	0.58	0.107*
9	49°20′53.5″N, 0°41′03.2″W	15	0.43	0.47	0.094	1	–	–	–

Less intensive surveys were more recently conducted further West and East in order to check for additional mixed or pure *J. albifrons*/*J. praehirsuta* populations (such populations were found but not analyzed in this study, see 3).

Second, we searched for similarly mixed populations of the same pair of species in a region where no hybridization had been found despite extensive field studies (area around Roscoff biology station, Brittany, France, Figure 2, Bocquet & Solignac, 1969; Solignac, 1969b). In this region, we selected six sampling sites where the two species were found at the same location (Figure 2 and Table 1).

In both regions (Brittany and Normandy), we looked for individuals of the *Jaera albifrons* complex under rocks and on seaweeds (*Ascophyllum nodosum*,* Fucus vesiculosus,* and *Fucus serratus* essentially) in the intertidal zone. Animals found on rocks were collected in the field using a small brush. By contrast, samples of seaweeds were brought back to the laboratory where we checked for the presence of *Jaera* individuals by shaking algae repeatedly in freshwater (Solignac, 1978). All individuals where kept alive until identification based upon observation of male secondary sexual traits. Because females of all species are morphologically identical, this study is based on male individuals only. As adult females are larger than males, many females could be left alive in the field. All collected individuals were fixed in ethanol after species identification.

Finally, one sampling site (Ste‐Honorine‐des‐Pertes, site 8 in Figure 2) was selected for a detailed analysis of the micro‐distribution of individuals. In this site, we performed an exhaustive survey on a large portion of the beach, recording the precise localization of each individual with a Trimble GeoExplorer 6000 GPS (average horizontal accuracy 55 mm). There we also collected females, which were kept in the laboratory until they produced offspring (sperm storage allows females to produce offspring in the absence of males). These offspring were reared in the laboratory for at least 6 weeks, until each individual could be sexed and each male could be identified based on secondary sexual traits. This identification of series of male sibs gave a presumptive species identification for their mother (e.g., a female could be classified as *J. albifrons, J. praehirsuta,* or “hybrid” if it gave birth to a series of males bearing *J. albifrons*,* J. praehirsuta* or intermediate morphological traits, respectively).

### 2.3 Genetic analyses

All genetic analyses are based on individual genotypes obtained at 23 microsatellite loci (all loci described in Ribardière, Broquet, & Daguin‐Thiebaut, 2015). DNA extraction and amplification followed the protocols described in Ribardière et al. (2015). Pairwise linkage disequilibrium between loci was tested in fstat version 2.9.3.2 (Goudet, 2001) in order to avoid redundant information. Departure from Hardy–Weinberg equilibrium was also tested in fstat in order to detect technical artifacts (null alleles or allelic dropouts) or departure from random mating within sampling sites. The occurrence of null alleles, already detected in the *Jaera albifrons* complex with these microsatellites (Ribardière et al., 2015), was specifically investigated with the software microchecker (Van Oosterhout, Hutchinson, Wills, & Shipley, 2004). About 10% of the genotypes where randomly replicated in order to evaluate the frequency of genotyping errors and for each locus genotyping error rate was calculated as error rate = (number of false genotypes)/(total number of repeated genotypes). The level of polymorphism was estimated by measuring observed and expected heterozygosity in fstat.

Our test of reproductive isolation or hybridization between *J. albifrons* and *J. praehirsuta* within our two sampling regions (Brittany and Normandy) is based upon estimates of genetic differentiation. We estimated the distribution of genetic variance among sampling sites within a species (*F*_SC_) and between species (*F*_CT_) in a hierarchical analysis of molecular variance (AMOVA, Excoffier, Smouse, & Quattro, 1992) implemented in arlequin version 3.5 (Excoffier & Lischer, 2010). We performed this analysis independently within each region. The between‐species component *F*_CT_ will thus inform us on the strength of genetic differentiation between *J. albifrons* and *J. praehirsuta* within each region. Small samples were not included in these analyses (i.e., individuals with an intermediate phenotype, and individuals from site 9, where one of the two species was represented by only one individual). These analyses were performed using both allelic frequencies (F_ST_‐like) and the distance between alleles (R_ST_‐like), and significance was assessed using 10,100 permutations as implemented in arlequin. We ran these analyses first using all markers (global AMOVA) and then for each microsatellite locus independently (locus‐by‐locus AMOVA).

Because some loci showed strongly contrasted levels of between‐species genetic structure (*F*_CT_) in Normandy vs. Brittany (see 3), the significance of the difference between *F*_CT‐Normandy_ against *F*_CT‐Brittany_ was tested by bootstrapping individuals 1,000 times in R version 3.2.3 (R Core Team, 2016). This allowed us to estimate how often the two *F*_CT_ values obtained from a given resampled dataset overlapped, and thus, whether *F*_CT‐Normandy_ differed significantly from *F*_CT‐Brittany_ at the locus tested.

Pairwise estimates of genetic differentiation between samples were also obtained in a nonhierarchical model in arlequin (i.e., *F*_ST_ between all pairs of populations, where a population is defined by a given species in a given sampling site, Table 1). This is useful (1) to evaluate whether the global differentiation between species is consistent across sampling sites (i.e., using *F*_ST_ between species within each site separately), and (2) to investigate genetic structure within each species separately, in particular by testing for isolation by distance between sampling sites with spagedi 1.4 (Hardy & Vekemans, 2002) and genepop 4.2.2 (Rousset, 2008).

Finally, the distribution of genetic variance was also investigated using individual analyses without a priori grouping of samples. We first ran a clustering analysis within each region using structure 2.3.4 (Pritchard, Stephens, & Donnelly, 2000) with an admixture model (10 independent repetitions, burn‐in period = 50,000, MCMC = 300,000). The most likely number of clusters (*K*) was determined *via* harvester v.0.6.1 (Earl, 2012) using Δ*K* as described by Evanno, Regnaut, and Goudet (2005). The *Ancestdist* option was used in STRUCTURE to calculate 95% probability intervals for an individual's membership *q* to each cluster. The width of such intervals (difference between upper and lower bounds) gave us an estimate of the precision of membership values. Second, we performed a principal component analysis (PCA) based on all individual genotypes using the R package ade4 (Dray & Dufour, 2007). Because the locus‐by‐locus AMOVA showed that two loci had a striking behavior (see 3), we ran both analyses (PCA and structure) with and without these two loci.

## 3 RESULTS

### 3.1 Population survey

In Brittany, we sampled 142 *J. albifrons* and 80 *J. praehirsuta* in six sites where the two species were co‐occurring (Figure 2 and Table 1, only males are considered throughout the paper, unless stated otherwise). In these sites, all *J. albifrons* but two were found under rocks, while all *J. praehirsuta* except three were found on seaweeds (located directly above rocks or within a radius of a few meters). In this region, all individuals could be morphologically identified at the species level without any overlap of traits (i.e., no individuals with intermediate morphology).

In Normandy, we sampled 44 *J. albifrons* and 57 *J. praehirsuta* from the three mixed populations that we found (sites 7–9, Figure 2). Contrary to the Brittany situation, all individuals were collected under rocks, while no individuals could be found on seaweeds. In addition, we found nine individuals clearly showing intermediate morphological traits as described by Solignac (Figure 1, “hybrid types” 5–13 in Solignac, 1978; p.172–177). These individuals will be referred to as “intermediate phenotypes” hereafter. The portion of the coast that was intensively surveyed revealed a single pure *J. albifrons* population located >70 km East of mixed populations (empty circle in Figure 2).

Later, less intensive surveys revealed another pure *J. albifrons* population 30 km West (Figure 2), and interestingly, one additional mixed *J. albifrons*/*J. praehirsuta* population (with intermediate phenotypes) further East (location Yport, Figure 2). No *J. praehirsuta* individuals were found outside of mixed populations anywhere in Normandy.

The fine‐scale distribution of individuals at site 8 (Ste‐Honorine‐des‐Pertes, Normandy) showed that individuals (61 males and 138 females) of the two species and intermediate phenotypes were largely intermingled, with *J. praehirsuta* being distributed all along the shore while the distribution of *J. albifrons* was more irregular (Figure S1).

### 3.2 Genetic diversity

All microsatellite loci could be amplified in individuals of the two species (consistent with Ribardière et al., 2015) as well as in individuals with intermediate phenotypes. There was no linkage disequilibrium after Bonferroni's correction between all pairs of loci in each population, so that all 23 markers were kept for further analyses. The level of polymorphism was globally consistent across species (Table 1). Unless stated otherwise, individuals with intermediate phenotypes were removed from the following analyses for we had too few of them (2 in Grandcamp, site 7, and 7 in Ste‐Honorine‐des‐Pertes, site 8, Figure 2). Observed heterozygosity *H*o (0.45 in *J. albifrons* and 0.51 in *J. praehirsuta*) was on average lower than within population gene diversity *He* (0.51 and 0.59, respectively), resulting in a significant departure from Hardy–Weinberg equilibrium (*F*_IS_ values in Table 1). Departure from HWE was driven in Brittany by loci Ja37, Ja39, Ja55, and Ja94, and in Normandy by locus Ja55 (loci with significant positive *F*_IS_ in one to six samples, data not shown). These loci, except Ja94, showed signs of a null allele in more than half of populations, as tested using microchecker. While we did not detect locus‐specific HW disequilibrium patterns that were consistent across populations, the main downstream quantitative analysis (analysis of molecular variance) was run with and without the four loci cited above (and we report locus‐specific results as well). Genotyping error rates estimated from replicated individuals ranged from 0% to 5.88% per locus (average 1.7%) and were due roughly equally to allelic dropouts and false alleles.

### 3.3 Genetic differentiation between *J. albifrons* and *J. praehirsuta*

**Figure 3:**
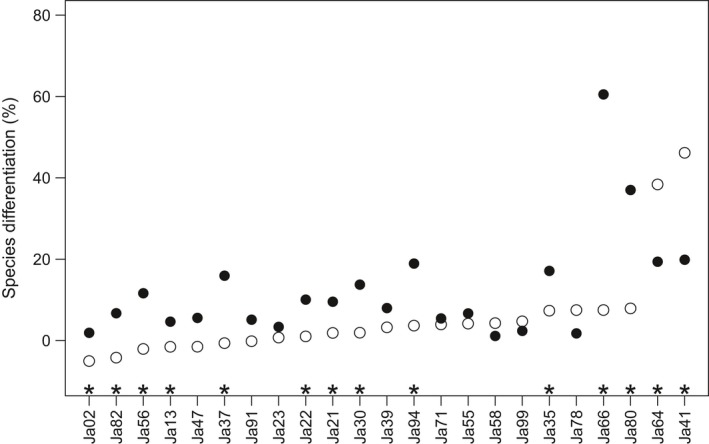
Locus‐by‐locus genetic differentiation between *J. albifrons* and *J. praehirsuta* (*F*_CT_, expressed in this figure as the percentage of genetic variation due to differences between species) in Brittany (black dots) and in Normandy (white dots). Significant differentiation between *F*_CT__‐Brittany_ and *F*_CT__‐Normandy_ is represented by a star above the locus name. Here the loci are arranged by increasing order of *F*_CT__‐Normandy_. We see that all white dots but two lie near zero (no differentiation between species in Normandy) while the two remaining loci (Ja41 and Ja64) show a strong differentiation. These two microsatellites are the only loci showing a significantly stronger differentiation between species in Normandy than in Brittany (white vs. black dots). In Brittany, the average level of differentiation between species is near 13% and there is also heterogeneity across loci

The hierarchical analysis of molecular variance based on 23 microsatellite loci (Table 2) showed that the between‐species differentiation was higher in Brittany (*F*_CT‐Brittany_ = 0.132, *p *< .005) than in Normandy (*F*_CT‐Normandy_ = 0.074, *p *= .34). Accordingly, the locus‐by‐locus AMOVA showed that most loci were less differentiated between species in Normandy than in Brittany (Figure 3). However, two loci (Ja41 and Ja64) revealed a strikingly different pattern: These two loci showed a very strong level of differentiation (Ja41, *F*_CT‐Normandy_ = 0.462; Ja64, *F*_CT‐Normandy_ = 0.384) in Normandy region while the remaining 21 loci showed no or little differentiation (locus specific *F*_CT‐Normandy_ ranged from −0.05 to 0.079], Figure 3). Without these two peculiar loci, there is thus no genetic differentiation between species in Normandy (*F*_CT‐Normandy_ = 0.017, *F*_CT‐Brittany_ = 0.125, Table 2). Note that this result is unchanged when removing the four loci that showed a departure from HW equilibrium in some populations (not shown).

**Table 2:** Distribution of the genetic variation estimated through hierarchical analyses of molecular variance in regions Brittany and Normandy (as defined in Figure 1). We present the results obtained with and without loci Ja41 and Ja64, which show a very strong differentiation in Normandy (see *F*_CT_ at these two loci)

Source of variation	Brittany			Normandy		
	% of total variation	*F*‐stat	*p*‐Value	% of total variation	*F*‐stat	*p*‐Value^a^
23 loci						
Among sampling sites						
Between species	13.2	*F*_CT_ = 0.132	.001	7.4	*F*_CT_ = 0.074	*.336*
Within species	1.3	*F*_SC_ = 0.015	<.001	1.5	*F*_SC_ = 0.016	.003
Within sampling sites						
Among individuals	9.7	*F*_IS_ = 0.114	<.001	8.8	*F*_IS_ = 0.097	<.001
Within individuals	75.8		<.001	82.3		<.001
21 loci (without Ja41 & Ja64)						
Among sampling sites						
Between species	12.5	*F*_CT_ = 0.125	.002	1.7	*F*_CT_ = 0.017	***.328***
Within species	1.2	*F*_SC_ = 0.013	<.001	1.8	*F*_SC_ = 0.019	**.001**
Within sampling sites						
AMONG individuals	10.1	*F*_IS_ = 0.117	<.001	8.4	*F*_IS_ = 0.087	<.001
Within individuals	76.3		<.001	88.1		<.001
2 loci (Ja41 and Ja64)						
Among sampling sites						
Between species	20.2	*F*_CT_ = 0.202	.003	42.8	*F*_CT_ = 0.428	*.332*
Within species	2.5	*F*_SC_ = 0.032	<.001	−0.7	*F*_SC_ = −0.012	.65
Within sampling sites						
Among individuals	8.9	*F*_IS_ = 0.115	<.001	11.6	*F*_IS_ = 0.2	<.001
Within individuals	68.4		<.001	46.3		<.001

a The statistical significance of between‐species variation was tested using permutations of sites between species, which is essentially powerless in region Normandy where only two sites harboring mixed populations were found. The relevant *p*‐values (indicated in italic) are thus meaningless, and the differentiation between species was better tested in this case using nonhierarchical *F*‐statistics within each site (see text).

Moreover, the differentiation between species at loci Ja41 and Ja64 in Normandy was not only much stronger than at other loci but it was also stronger than the differentiation observed at the same two loci in Brittany (Ja41, *F*_CT‐Brittany_ = 0.199, Ja64, *F*_CT‐Brittany_ = 0.194, Figure 3), and this difference was significantly different from 0 (bootstrap *p*‐value <.001 for Ja41 and *p *= .001 for Ja64, Figures S2 and S3). The distribution of allelic frequencies at these two loci is presented in Figures S4 and S5.

Although the heterogeneity across loci appeared somewhat lower in Brittany (Figure 3), there was nonetheless some variation, with two other loci showing a particularly strong level of differentiation (Ja66, *F*_CT‐Brittany_ = 0.605; Ja80, *F*_CT‐Brittany_ = 0.37). The difference in *F*_CT_ between regions was significant at these two loci (bootstrap *p*‐value < .001 for Ja66 and Ja80, Figure S3).

Because we studied only three mixed populations in Normandy, and one of them contained nearly only *J. albifrons* (site 9, Longues‐sur‐Mer, Figure 2, Table 1), the permutation procedure implemented in arlequin to test for the significance of *F*_CT‐Normandy_ is essentially powerless (the between‐species component of genetic variation is tested by permuting populations within species). We therefore checked whether the patterns found in the AMOVA (which considers all sites simultaneously) were consistent across sites. Tables 3 and 4 show pairwise *F*_ST_ values calculated in a simple nonhierarchical framework. Most importantly, it shows that the between‐species differentiation was consistent across sites, both in Normandy (*F*_ST_ between species equal to 0.104 at Grandcamp, site7, and 0.101 at Ste‐Honorine‐des‐Pertes, site 8) and in Brittany (*F*_ST_ between species within sites in [0.1; 0.19]). These results consider all loci, but the same geographical consistency is observed when considering the locus‐specific patterns described above (data not shown). That is, the global AMOVA results are repeatable across sites (e.g., *F*_ST_ at the two sites from Normandy = 0.546 (site 7) and 0.396 (site 8) when considering only loci Ja41 and Ja64, and *F*_ST_ = 0.05 (site 7) and 0.056 (site 8) with all other loci).

**Table 3:** Pairwise genetic differentiation between populations in Brittany. Above diagonal: pairwise *F*_ST_. Below diagonal: exact test of population differentiation *p*‐value (in bold when significant). Values in the gray area correspond to inter‐specific *F*_ST_

	*J. albifrons*						*J. praehirsuta*					
	1	2	3	4	5	6	1	2	3	4	5	6
*J. albifrons*												
1	–	0.02	0.061	0.06	0.035	0.034	0.178	0.161	0.14	0.14	0.062	0.167
2	**0.003**	–	0.066	0.055	0.036	0.05	0.175	0.151	0.137	0.141	0.059	0.161
3	**0**	**0**	–	0.005	0.021	0.017	0.182	0.177	0.155	0.149	0.099	0.187
4	**0**	**0**	0.190	–	0.015	0.024	0.174	0.167	0.141	0.14	0.092	0.179
5	**0**	**0**	**0.003**	**0.014**	–	0.009	0.207	0.195	0.17	0.166	0.1	0.196
6	**0**	**0**	**0.008**	**0.001**	0.116	–	0.201	0.184	0.157	0.152	0.103	0.19
*J. praehirsuta*												
1	**0**	**0**	**0**	**0**	**0**	**0**	–	−0.007	0.01	−0.005	−0.048	0.017
2	**0**	**0**	**0**	**0**	**0**	**0**	0.671	–	−0.005	−0. 004	−0.068	0.008
3	**0**	**0**	**0**	**0**	**0**	**0**	0.160	0.715	–	−0.008	−0.061	0.004
4	**0**	**0**	**0**	**0**	**0**	**0**	0.737	0.657	0.919	–	−0.074	0.005
5	**0.004**	**0.003**	**0**	**0**	**0**	**0**	0.979	0.988	0.999	0.999	–	−0.075
6	**0**	**0**	**0**	**0**	**0**	**0**	**0.042**	0.278	0.295	0.268	1	–

**Table 4:** Pairwise genetic differentiation between populations in Normandy. Above diagonal: pairwise *F*_ST_. Below diagonal: exact test of population differentiation *p*‐value (in bold when significant). Values in the gray area correspond to inter‐specific *F*_ST_

	*J. albifrons*		*J. praehirsuta*	
	7	8	7	8
*J. albifrons*				
7	–	0.025	0.104	0.073
8	**0.037**	–	0.153	0.101
*J. praehirsuta*				
7	**0**	**0**	–	0.018
8	**0**	**0**	**0.015**	–

**Figure 4:**
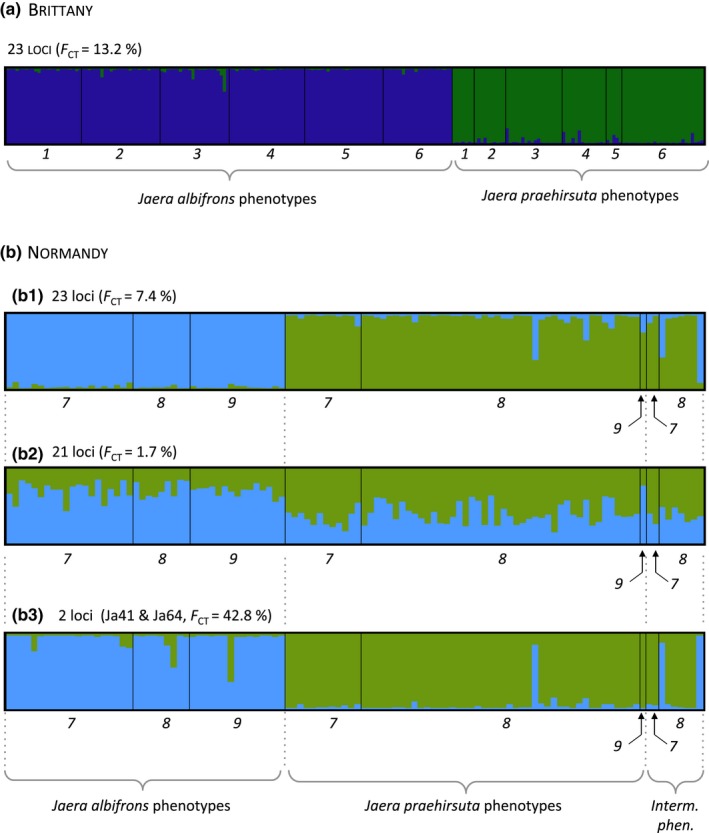
structure clusters (K = 2) defined in Brittany (a) and Normandy (b). Because loci Ja41 and Ja64 are outliers in Normandy (see text and Figure 3), structure results for this region are presented with all loci (23 loci, panel b1), without the two outliers (21 loci, panel b2), and considering only Ja41 and Ja64 (2 loci, panel b3). Numbers refer to sampling sites (Figure 2). We see that individuals morphologically identified as *J. albifrons* or *J. praehirsuta* cluster into two distinct groups in Brittany (regardless of sampling location, panel a), while this will remain true in Normandy only due to the effect of two markers of twenty‐three (panels B1, B2, and B3)

Individual analyses bring some complementary information, in particular because the individuals with intermediate morphology could be included in spite of their low abundance (as well as individuals from site 9). Running structure with K = 2, we found that *J. albifrons* and *J. praehirsuta* cluster into two clearly identified groups both in Brittany and Normandy using a panel of 23 loci (Figure 4). However, while an individual's membership *q* to its assigned cluster was similar for both species in Brittany and Normandy (membership averaged over all individuals and 10 structure runs, Brittany: ${\overset{¯}{q}}_{\mathrm{albifrons}}$ = 0.99, ${\overset{¯}{q}}_{\mathrm{praehirsuta}}$ = 0.97, and Normandy: ${\overset{¯}{q}}_{\mathrm{albifrons}}$ = 0.97, ${\overset{¯}{q}}_{\mathrm{praehirsuta}}$ = 0.95), the uncertainty associated with *q* was larger in Normandy (average width of 95% probability interval $w_{\mathrm{albifrons}}^{95}$ = 0.24, $w_{\mathrm{praehirsuta}}^{95}$ = 0.32) than Brittany ($w_{\mathrm{albifrons}}^{95}$ = 0.12, $w_{\mathrm{praehirsuta}}^{95}$ = 0.19). As it turned out, the apparent genetic clustering in Normandy was almost entirely due to the effect of two loci only (Ja41 and Ja64), while a structure analysis using the remaining 21 loci showed that the two species were genetically homogeneous (Figure 4b).

**Figure 5:**
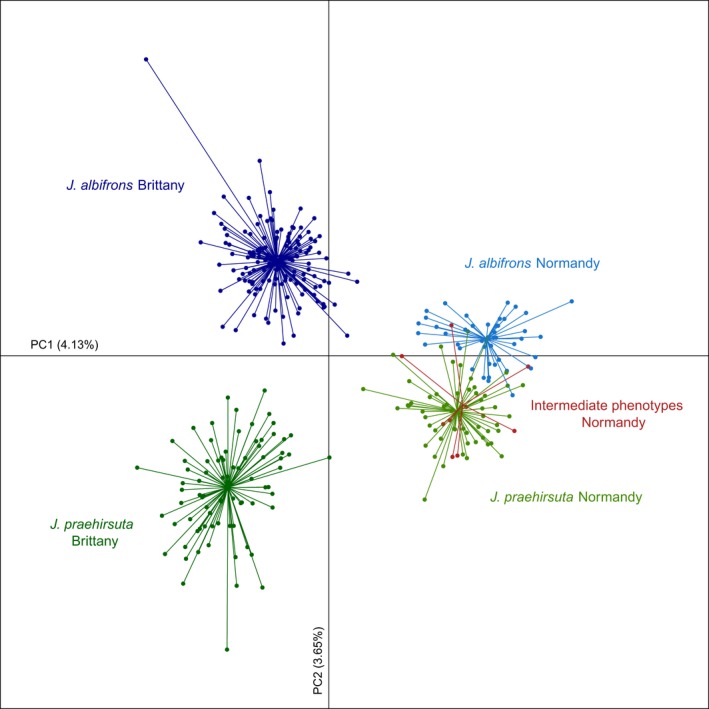
Principal component analysis based on individual multilocus genotypes at 23 microsatellite loci. The first axis separates individuals from Brittany (left) and Normandy (right). The second axis shows the genetic divergence between males bearing sexual traits typical of *J. albifrons* vs. *J. praehirsuta*. We see that these two types of males are less genetically differentiated in region Normandy (in agreement with results from the analysis of molecular variance and structure results from Figure 4) and males with an intermediate phenotype are undifferentiated from *J. praehirsuta*

The results from molecular analyses of variance and clustering analyses could be well visualized using PCA performed with all 23 loci (Figure 5). The two isopod species were clearly differentiated in one region (Brittany) and less so in the other one (Normandy). We see also that individuals with intermediate phenotypes were genetically indistinguishable from individuals with *J. praehirsuta* traits.

The particular geographical distribution of individuals (replicates of mixed populations comprising intermediate phenotypes and absence of clinal structure, see 4) precluded the use of genetic tools dedicated to the analysis of hybridization in clinal hybrid zones.

### 3.4 Genetic structure within species

Because there is ongoing hybridization and introgression between the two species in Normandy (see 4), the within‐species genetic structure is best investigated using samples from Brittany. Brittany is also the region where we have more sampling locations. The genetic structure among *J. albifrons* samples appeared slightly higher than within *J. praehirsuta*. This is visible from pairwise *F*_ST_ estimates (Table 3), ranging from 0.005 to 0.066 (13 of 15 pairs significantly different) when considering only *J. albifrons* within Brittany, and −0.075 to 0.017 (one of 15 pairs significant) when considering only *J. praehirsuta*.

In addition, a significant pattern of isolation‐by‐distance (Figure S6) was observed in Brittany both in *J. albifrons* (*R* ^2^ = 0.73, Mantel test *p*‐value < .01) and in *J. praehirsuta* (*R* ^2^ = 0.11, *p*‐value = .02). In line with the pairwise *F*_ST_ results, genetic differentiation increased more rapidly with distance in *J. albifrons* than in *J. praehirsuta*, although 95% confidence intervals calculated in genepop overlapped (10,000 permutations, *J. albifrons* [0.013, 0.037], *J. praehirsuta* [−0.0005, 0.018], Figure S6).

## 4 DISCUSSION

The first result of this study is the clear‐cut confirmation that introgressive hybridization is happening between *J. albifrons* and *J. praehirsuta* in at least two mixed populations from Normandy, France. As developed below, this opens interesting questions regarding the conditions of coexistence of the two parental morphs in hybridizing populations that seem to receive no influx from pure parental populations and shows no detectable ecological heterogeneity.

### 4.1 Hybridization between *Jaera albifrons* and *J. praehirsuta*

Analyses of molecular variance (Table 2 and Figure 3) and admixture analyses (Figures 4 and 5) both showed that mixed populations from Normandy have a homogeneous genetic structure at 21 of 23 multi‐allelic loci. Critically, this genetic homogeneity contrasts with the differentiation observed in mixed populations from Brittany, where individuals bearing sexual traits specific to *J. albifrons* or *J. praehirsuta* cluster into clearly marked genetic groups (Figures 4a and 5). Hence, shared ancestral polymorphism cannot explain the lack of differentiation between species in Normandy, which therefore supports the hypothesis of ongoing hybridization.

These findings agree with the conclusions reached by C. Bocquet and M. Solignac nearly 50 years ago, who studied the morphological variation of secondary sexual traits in a population from the same region (Luc‐sur‐Mer, Figure 2, Bocquet & Solignac, 1969; Solignac, 1969a,b, 1978 chapter 6). Similarly to the results reported by these authors, we found that in Normandy, several males have intermediate phenotypes and the two species occupy the same habitat (under stones and pebbles on the shore) while in Brittany we did not detect any intermediate phenotypes and the two species occupy two different habitats, with some overlap; *J. albifrons* lives primarily under stones, while *J. praehirsuta* is found primarily on seaweeds.

We conclude from these observations and the contrast in species divergence in the two regions that the two species are currently hybridizing in populations from Normandy, but not in Brittany.

### 4.2 A semi‐permeable barrier to gene flow

The genetic homogeneity observed across species in Normandy further shows that hybridization has been introgressive, as correctly concluded by Solignac (1969b) from the continuous range of morphological characteristics observed in natural populations and by comparison with experimental crosses (Bocquet & Solignac, 1969). While it is now clearly established that introgression proceeds differentially across loci in hybrid zones (with no known exceptions, Harrison & Larson, 2014), investigating this variation was not part of our original plan with this study given that we were using a panel of only 23 loci. However, the locus‐by‐locus AMOVA analyses revealed a surprisingly trenchant pattern, whereby 21 loci showed no differentiation at all between species in populations from Normandy (*F*_CT_ in [−0.05; 0.08]) and the two remaining loci where strongly differentiated (*F*_CT_ = 0.384 and 0.462). Moreover, these two loci were also significantly more differentiated in the hybridizing populations (Normandy) than in reproductively isolated ones (Brittany) while showing no differentiation within each species (see *F*_SC_ values in Table 2). This strongly suggests that there is a semi‐permeable barrier to gene flow between *J. albifrons* and *J. praehirsuta* in hybridizing populations from Normandy. Hypotheses other than a reduction in gene flow at these loci seem impossible to reconcile with the fact that the same two loci are significantly less differentiated in nonhybridizing sympatric populations. Alternate hypotheses such as the differential sorting of ancestral polymorphism or reduced variability at these loci due to a locally low recombination rate would require a history of differentiation between species whereby ancestral polymorphism or recombination have evolved differentially in the two regions studied (ca 250 km apart). While this is theoretically possible, a more parsimonious hypothesis is that the two loci are encompassed in one or two genomic regions where interspecific gene flow is hampered because these regions are linked with one or several isolating barriers.

Additional indicators of a semi‐permeable barrier to gene flow are two other markers (Ja66 and Ja80) also showing a heterogeneous pattern. As most markers, they are more differentiated in Brittany than in Normandy, but, interestingly, they show a stronger differentiation than the other loci in Brittany (Figure 3). We cannot currently make assumptions based only on these results, which emphasize the necessity to study the heterogeneity of genome with an extended set of genetic markers.

### 4.3 Is introgression symmetrical?

Interspecific crosses are generally not equally likely in both directions, especially when behavioral isolation is involved (e.g., Coyne & Orr, 2004, p. 226). Such asymmetries leave specific signatures in the genome that are most easily detected by comparing uni‐ and bi‐parentally inherited genetic variation (typically, markers from the mitochondrial and nuclear DNA, e.g., Toews & Brelsford, 2012). It would be interesting to test for asymmetric introgression of mtDNA in our system given that Bocquet and Solignac (1969) have suggested that interspecific crosses may occur more easily in one direction (female *J. praehirsuta* x male *J. albifrons*) than the other. This result was obtained from experimental crosses with individuals from a hybridizing population (Luc‐sur‐Mer, Normandy), and the asymmetry was further confirmed by Solignac (1981) using individuals from other origins (nonhybridizing populations). Yet we could not use this approach here because mtDNA analyses so far have indicated that most of the genetic variation is shared by all species of the *Jaera albifrons* complex (perhaps excluding the American species *J. posthirsuta*, which has not been included in these analyses). That is, the four European species form a polyphyletic clade (16S rDNA, Mifsud, 2011; and COI, A. Ribardière and T. Broquet, unpublished), in strong contrast with the patterns obtained with nuclear data (AFLP, Mifsud, 2011; and microsatellites, this study). There are no mitochondrial haplotypes or clades that are specific to *J. albifrons* or *J. praehirsuta* (not shown), and the cyto‐nuclear discordance, also certainly interesting in its own right, is not informative of recent introgression directionality. The symmetry of introgression can in some cases be evaluated by taking advantage of differences between nuclear loci (differential introgression), but our microsatellite dataset is too limited for this approach. Nevertheless, results of admixture analyses show that individuals showing an intermediate phenotype share more genetic background with *J. praehirsuta* (Figures 4 and 5) which suggests that introgression is asymmetric (genetic variation from *J. albifrons* introgressing into the genetic background of *J. praehirsuta*). This can also be seen by looking at allelic frequencies at loci Ja41 and Ja64 for the phenotypically intermediate individuals, which are similar to that of individuals bearing *J. praehirsuta* traits and different from the frequencies observed in *J. albifrons* (Figures S4 and S5).

### 4.4 Geographical structure and persistence of hybridizing populations

In a 1969 paper, C. Bocquet and M. Solignac reported that many “morphological hybrids” have been observed during the preceding 15 years in their study area of Luc‐Sur‐Mer (Bocquet & Solignac, 1969). There is no more suitable habitat at this site, but Solignac (1978, p. 171) mentioned that “hybrids” had been found in Ste‐Honorine‐des‐Pertes, which is one of the two sites sampled in the present study (site 8). This means that hybridizing populations have persisted in Normandy for at least several decades. Moreover, during a recent additional survey aiming at extending the 95‐km coastline region studied here, we found intermediate phenotypes in a population located roughly 100 km East of the mixed populations studied here (location “Yport”, Figure 2). Even more unexpectedly, we detected a *J. albifrons*—*J. praehirsuta* mixed population with some intermediate male phenotypes in the Isles of Scilly, UK, an archipelago that is located more than 400 km away across the English Channel (Figure 2). This means that hybridization between these two species is probably much more widespread than previously thought (Solignac, 1969a,b, 1978). Perhaps more importantly, this also strongly suggests that hybridizing populations have been persisting for a long time.

In this study, we identified nine individuals (of 110 males found in Normandy) showing intermediate morphological traits. While this figure depends on what one recognizes as morphologically pure or intermediate individuals, the majority of males clearly show strict *J. albifrons* or *J. praehirsuta* sexual traits despite the extensive genetic introgression demonstrated here (bimodal hybrid zone, Jiggins & Mallet, 2000). We concur with Solignac's observation (1978, p. 188) that the coexistence of the two morphs in spite of introgressive hybridization is of great interest, and we discuss below the mechanisms that may allow this coexistence in the long term and in repeated areas. This part of the discussion will focus on hybridizing populations only (i.e., results from Normandy).

The closest population containing only *J. albifrons* that we found was located at more than 30 km from the hybridizing populations. Moreover, the mixed populations that we found in this region are geographically isolated from one another (there is most likely a discontinuity at least between sites 7 and 8, Figure 2). All species of the *Jaera albifrons* group have a direct development without a dispersive larval phase, and in Normandy they do not live on seaweeds, which could potentially drift across populations. Gravid females caught in the water column could occasionally be moved over a great distance, but we did not find any *J. praehirsuta* outside of the mixed *J. albifrons*/*J. praehirsuta* populations in Normandy. It is difficult to conduct an exhaustive survey over large areas for such small species and we could have missed pure *J. albifrons* (and perhaps even *J. praehirsuta*) populations at dispersal distance from our hybridizing populations, but we feel that it is unlikely. Given that these crustaceans do not have a dispersive larval phase, and given the patchy distribution of habitats, we infer from our surveys that hybridizing populations are replicated and patchily distributed.

The hybridizing populations analyzed in this study do not seem to be flanked by—or otherwise functionally connected to—pure parental populations. They seem to be independent replicates of hybridizing populations potentially distributed on a much larger geographical area than the one studied here (e.g., on the U.K. coast). The influx of individuals from pure parental populations of *J. praehirsuta* (and probably *J. albifrons*) is thus most likely not one of the forces acting to stabilize the system. This interpretation needs further testing (e.g., from additional surveys and analyses of spatial genetic structure), as if this hypothesis is confirmed, it would exclude dispersal‐dependent models of hybrid zones, chief among them the tension zone model (Barton & Hewitt, 1985), which relies on a balance between immigration of parental genotypes and selection against hybrids. An alternative hybrid zone model without immigration from parental populations involves ecological variation and an advantage of hybrids in intermediate habitats (Moore, 1977). This hypothesis seems also be excluded in our case because we were unable to detect any variation in habitat within hybridizing populations (there was no identifiable variation in the distribution of *J. albifrons*‐like, *J. praehirsuta*‐like, and morphologically intermediate individuals within a site). Other classical models are also inappropriate, for they combine the tension zone balance with ecological variation, either through environmentally induced selection against hybrids (Endler, 1977) or a patchy distribution of habitats favoring one or the other species (mosaic hybrid zones, Harrison & Rand, 1989). The literature is also rich in empirical studies of hybridizing populations that do not fit one of these classical models, but it seems that situations where species coexist in spite of extensive introgression (i.e., bimodal hybrid zones) most often involve either an income of individuals from pure parental populations or ecological variation and habitat specialization within hybrid zones (or both). When incompletely isolated species occupy different ecological niches, comparative analyses of replicate hybrid zones have made quite clear that the maintenance of bimodality is correlated with the opportunity for ecological specialization (e.g., Culumber et al., 2011; Gagnaire et al., 2013). In the *Jaera albifrons*/*J. praehirsuta* system, the two species are more differentiated in our populations from Brittany where they specialize in two different habitats (rocks vs. seaweeds) but, interestingly, parental forms coexist despite introgression in hybridizing populations in Normandy where there is probably no habitat specialization.

Assuming that there is no immigration from pure parental populations and no ecological variability, what evolutionary forces would allow pure *J. albifrons* and *J. praehirsuta* phenotypes to coexist in hybridizing populations? Past work suggests two strong candidates. First, the strongest isolating barrier between species of the *Jaera albifrons* complex is sexual isolation (Solignac, 1981). The courtship behavior of males (plus perhaps unknown male characteristics such as pheromone production or other unnoticed phenotypic variation) and female preference may still partially isolate *J. albifrons* from *J. praehirsuta* in hybridizing populations. One compelling hypothesis in this regard is that females of one species accept heterospecific mating more readily than females of the alternate species. This is nearly the rule in case of behavioral isolation (e.g., Coyne & Orr, 2004) and empirical tests suggest that this happens in the hybridizing populations studied here (although with a limited sample size, Bocquet & Solignac, 1969). The hypothesis that sexual isolation is a strong component is also in line with other examples where hybrid zones remain bimodal (reviewed in Jiggins & Mallet, 2000).

The second candidate is selection against hybrids. This hypothesis is supported by Solignac (1978, p. 186), who reported a strong hybrid breakdown in experimental F2 and backcrosses using individuals from the hybridizing population of Luc‐sur‐Mer, Normandy. This suggests that the *J. albifrons*/*J. praehirsuta* hybridizing populations documented in this study persist through a balance between hybridization versus partial (perhaps asymmetrical) sexual isolation and selection against certain recombined genotypes. This situation seems to be infrequent and the conditions of persistence for such a system deserve further inquiry. In particular, it is a potential empirical example of the model proposed by M'Gonigle, Mazzucco, Otto, and Dieckmann (2012), in which sexual selection, spatial variation in local carrying capacity, and female mate‐search costs allow partially divergent species to persist despite ecological equivalence. More generally, it may provide some insight into the debated role of sexual selection and sexual isolation in species divergence and coexistence. Yet it is remarkable that *J. albifrons* and *J. praehirsuta* coexist in the long term despite their seemingly ecological equivalence. It is possible that some unknown frequency‐dependent mechanism (e.g., via an action of pathogens or parasites) is acting to lower the likelihood of extinction of one or the two morphs.

### 4.5 Hybridizing and nonhybridizing mixed populations

Why do *J. praehirsuta* and *J. albifrons* hybridize when in contact in some populations and not in others? There are two obvious differences between our studied populations from Brittany and Normandy. First, where the two species were found to be nonhybridizing, they live on clearly different habitats (under rocks vs. on brown algae). The two habitats are located centimeters to meters away, and a few individuals of each species were found on the habitat favored by the other species, meaning that *J. albifrons* and *J. praehirsuta* meet each other frequently in these sites (i.e., why we call them mixed, or sympatric, populations: The two habitats are not separated by a large geographical distance and can be reached by dispersing individuals of either species). Yet these two habitats are radically different and are bound to impose a serious barrier to gene flow between species (ecological isolation). While neither species is restricted to one of these habitats in other parts of their range, the availability of distinct habitats may play an important role facilitating the coexistence of the two species and reducing hybridization opportunities. Appropriate seaweeds can also be found in hybridizing populations from Normandy, but they may not represent a suitable habitat there (e.g., because of wave exposure). Whatever the reason, there are no *Jaera* on algae in the hybridizing populations reported here. One plausible hypothesis is thus that ecological diversification facilitates the coexistence and divergence of *J. albifrons* and *J. praehirsuta* wherever this is possible. Another interesting idea, suggested by an anonymous reviewer, is that hybridization allows the hybrid and subsequently *J. praehirsuta* to acquire adaptations to the array of challenges presented in switching from a seaweed to a rocky habitat. One avenue of research to tackle these questions will be to search for pure *J. praehirsuta* populations occupying rocky habitats (and *J. albifrons* populations on seaweeds).

The second difference between hybridizing and reproductively isolated populations is their geographic location. Contrary to the idea that natural hybridization is the exception and not the rule in this system (Solignac, 1969a,b, 1978), we suggest that hybridizing populations may be found in a large geographic area. It is therefore possible that populations from two large geographic zones (one encompassing Normandy and the other one encompassing Brittany) have a distinct demographic and evolutionary history (e.g., with variations in the conditions of secondary contacts between species after periods of isolation). The intra‐specific genetic differentiation between regions appeared to be similar in *J. albifrons* and *J. praehirsuta* (*F*_ST_ = 0.14 and 0.15, respectively) and similar in intensity to the genetic differentiation between species observed in Brittany. This is also apparent on the PCA where the first axis partitions the genotypes in function of their geographic origin (Brittany on the left, Normandy on the right, Figure 5).

Testing these ideas will require analyzing the genetic structure of the two species over a large geographical scale and surveying mixed populations for morphological or genetic signs of hybridization in different habitat conditions.

## DATA ACCESSIBILITY

Multilocus genotypes at each sampling location are available in Dryad, https://doi.org/10.5061/dryad.st557.

## CONFLICT OF INTEREST

None declared.
