# Supplementary material for: Geographically distinct patterns of reproductive isolation and hybridization in two sympatric species of the Jaera albifrons complex (marine isopods)
Source: Ecol Evol. 2017 Jun 9;7(14):5352–65. doi: 10.1002/ece3.3106 (PMC5528242; doi:10.1002/ece3.3106)
Supplement: Supplementary file 1 [file ECE3-7-5352-s001.pdf]

Supplementary material

Figure S1: Distribution of individuals along the coast at Ste-Honorine-des-Pertes (site 8, Normandy) during a survey realized in summer 2014. Using a precise localization system (5.5 cm horizontal accuracy), we found that male individuals bearing sexual traits typical of *J. albifrons* or *J. praehirsuta* occupied the same microhabitats and show no particular distribution patterns (both species are intermixed with no apparent distributional gradient). Females were included in this analysis, and some of them were identified as *J. albifrons* or *J. praehirsuta* based on the sexual characters born by their sons (raised in the lab). For clarity purpose we did not distinguish males and females in this figure. Note also that many individuals are not visible here due to the near superposition of their locations.

Figure S2: Bootstrap distribution of F_{CT} (genetic differentiation between species) in Brittany (black) and Normandy (white) at loci Ja41 (Panel A) and Ja64 (B). Here we see that 1000 resampling iterations rarely produced a situation where $F_{CT\text{-Normandy}}$ was greater or equal to $F_{CT\text{-Brittany}}$ at these two loci (p-value ≤ 0.001). Observed F_{CT} are indicated by triangles (Brittany: black, Normandy: white).

Figure S3: Bootstrap distribution of F_{CT} (genetic differentiation between species) in Brittany (black) and Normandy (white) at 23 microsatellite loci. Observed F_{CT} in each region are indicated by triangles.

Figure S4: Allelic frequencies at locus Ja41 in *Jaera albifrons* (blue) and *Jaera praehirsuta* (green) from Brittany (panel A) and Normandy (panel B). Note that the F_{CT} values reported here were calculated in hierarchical analyses of variance without the individuals showing an intermediate phenotype (see main text).

Figure S5: Allelic frequencies at locus Ja64 in *Jaera albifrons* (blue) and *Jaera praehirsuta* (green) from Brittany (panel A) and Normandy (panel B). Note that the F_{CT} values reported here were calculated in

hierarchical analyses of variance without the individuals showing an intermediate phenotype (see main text).

Figure S6: Patterns of isolation-by distance observed in Brittany for *Jaera albifrons* (blue circles; y=0.0209x +0.0011, R^2 = 0.73, Mantel test *p*-value < 0.01) and *J. praehirsuta* (green circles; y=0.0065x -0.0133, R^2=0.11, Mantel test *p*-value = 0.02).

Figure S1

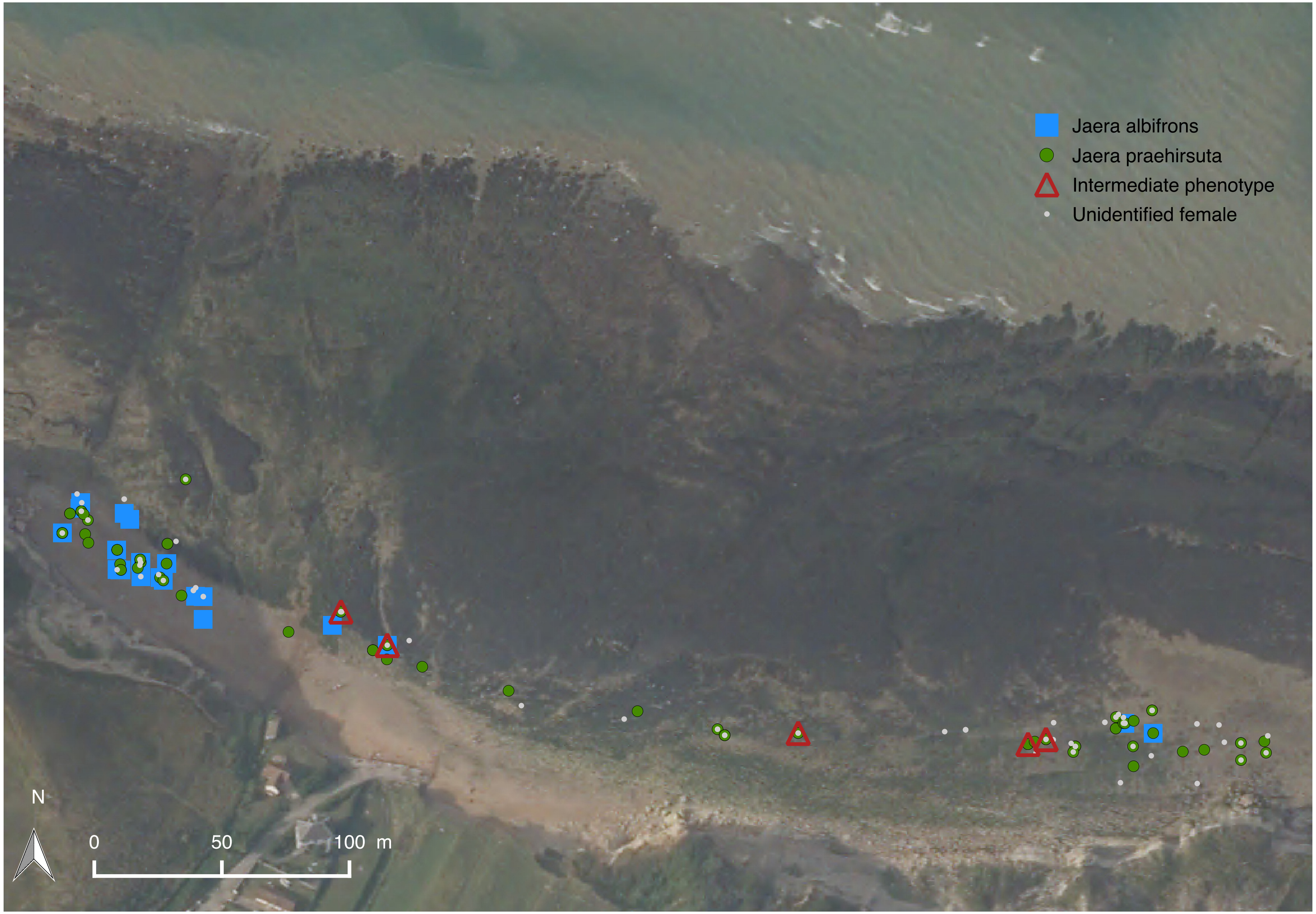

Jaera albifrons
Jaera praehirsuta
Intermediate phenotype
Unidentified female
N
0
50
100 m

Figure S2

A

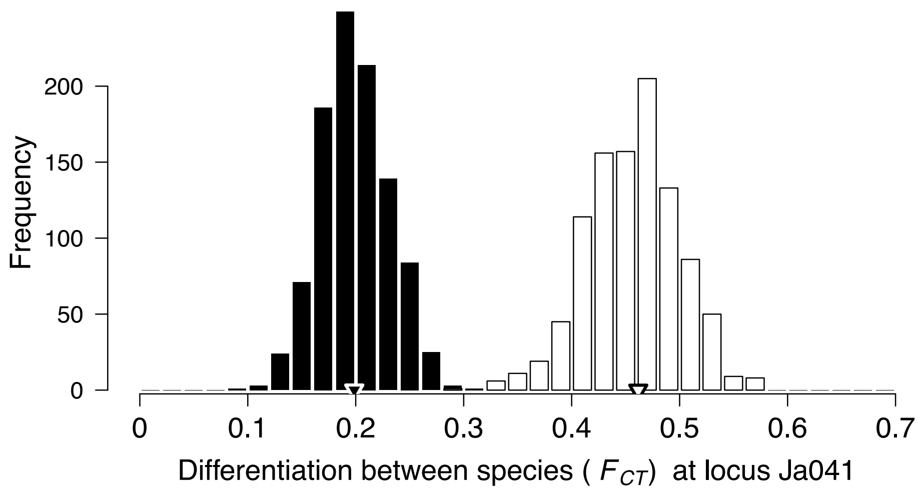
200
150
100
50
0
Frequency
0
0.1
0.2
0.3
0.4
0.5
0.6
0.7
Differentiation between species (F_{CT}) at locus Ja041

B

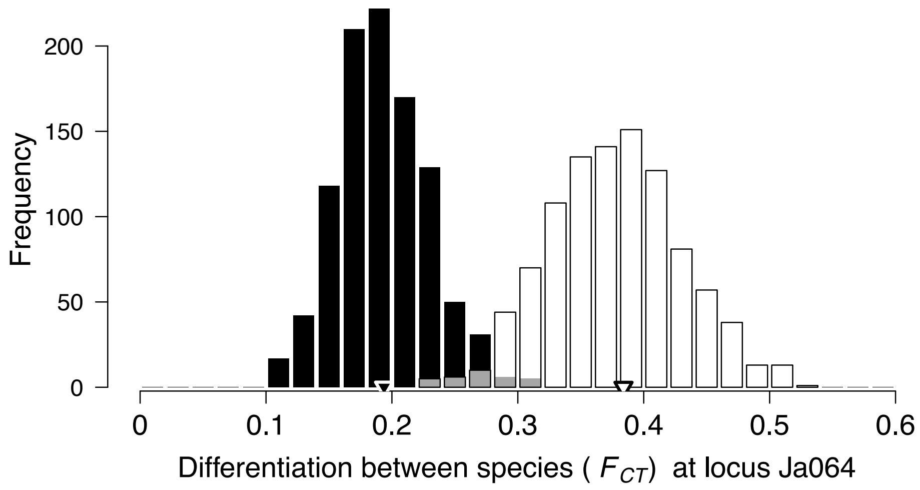
200
150
100
50
0
Frequency
0
0.1
0.2
0.3
0.4
0.5
0.6
Differentiation between species (F_{CT}) at locus Ja064

Figure S3

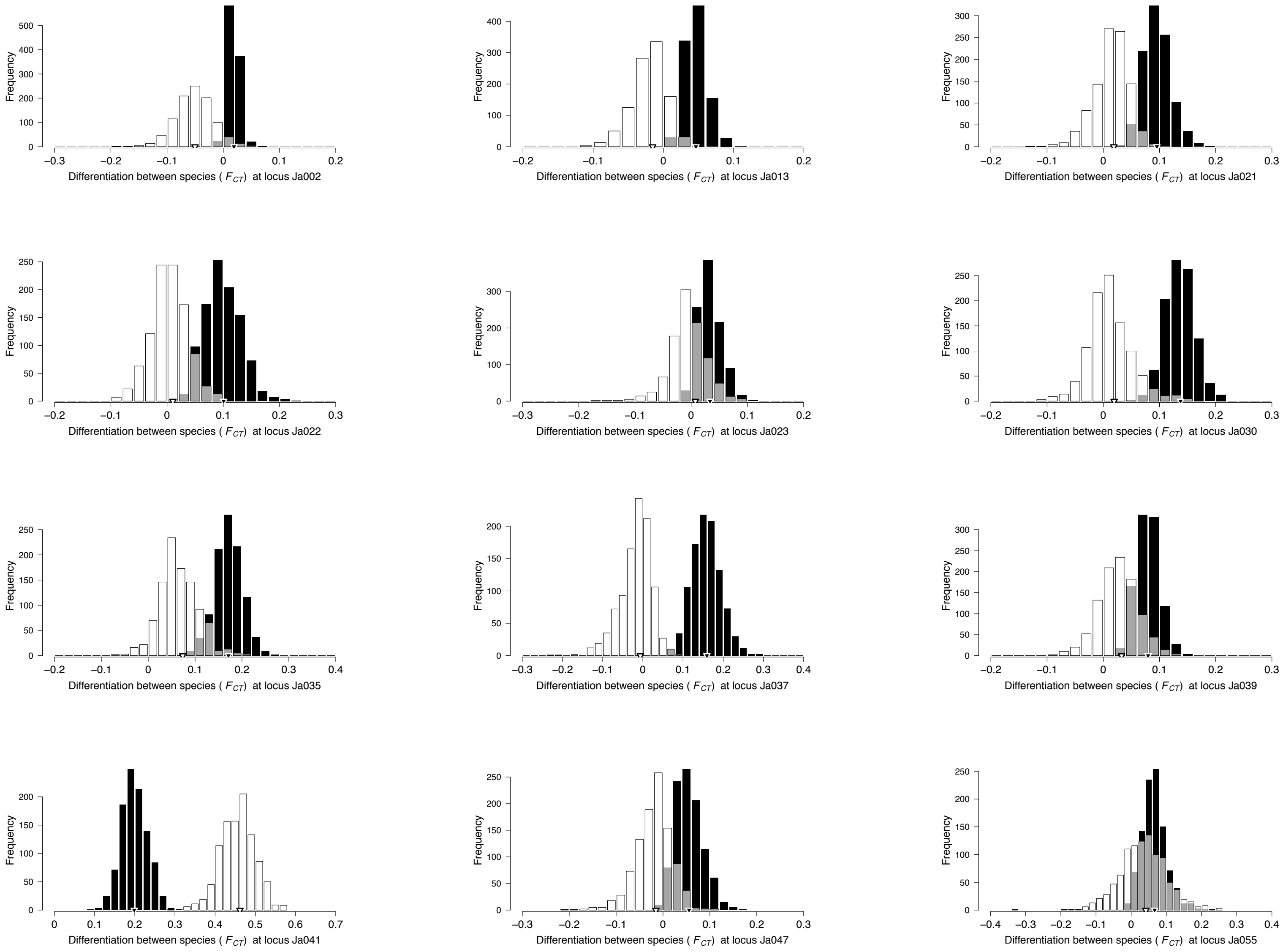

Frequency
Differentiation between species (F_{CT}) at locus Ja002
Differentiation between species (F_{CT}) at locus Ja013
Differentiation between species (F_{CT}) at locus Ja021
Differentiation between species (F_{CT}) at locus Ja022
Differentiation between species (F_{CT}) at locus Ja023
Differentiation between species (F_{CT}) at locus Ja030
Differentiation between species (F_{CT}) at locus Ja035
Differentiation between species (F_{CT}) at locus Ja037
Differentiation between species (F_{CT}) at locus Ja039
Differentiation between species (F_{CT}) at locus Ja041
Differentiation between species (F_{CT}) at locus Ja047
Differentiation between species (F_{CT}) at locus Ja055

Figure S3 (continued)

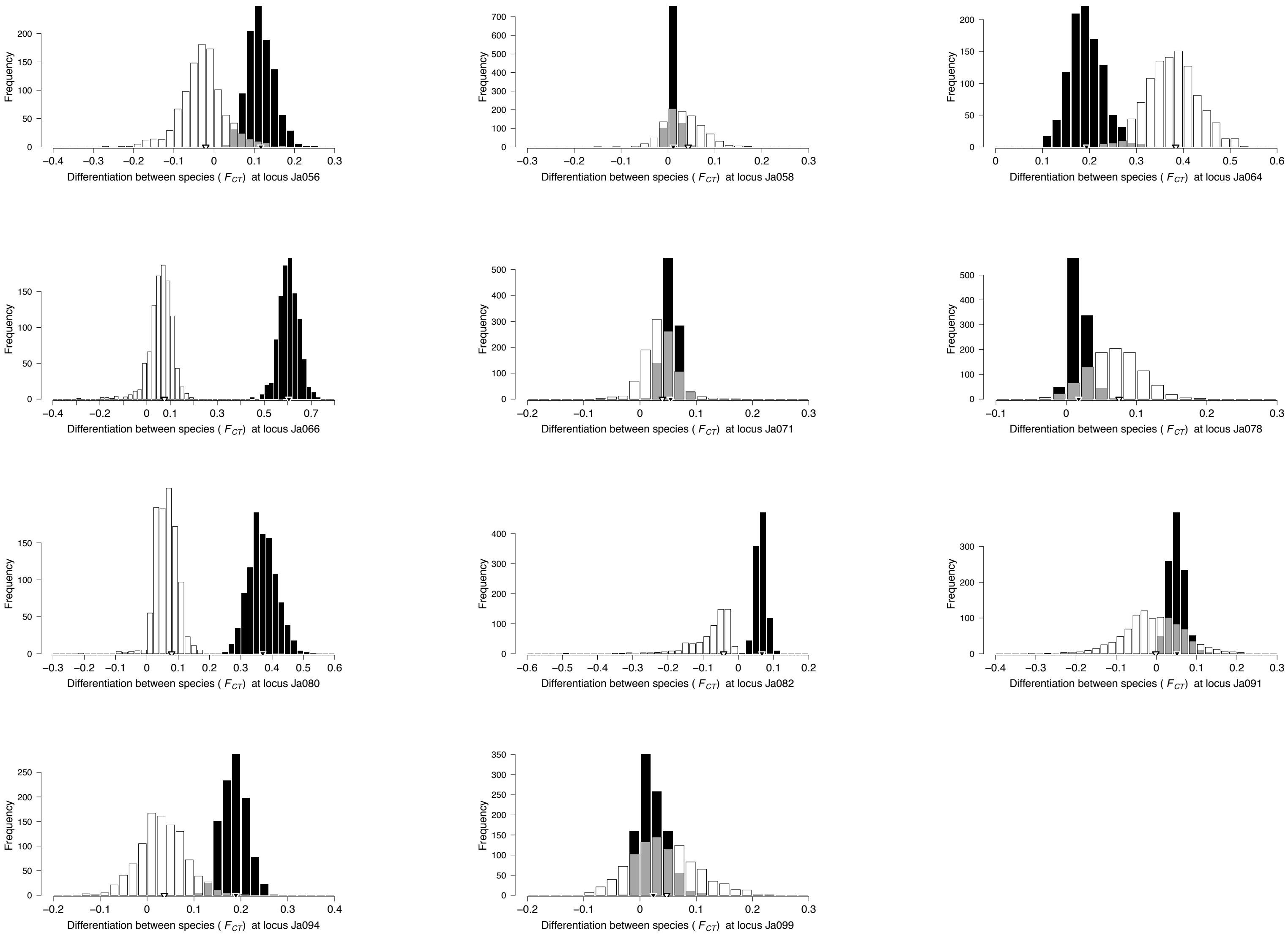

Figure S4

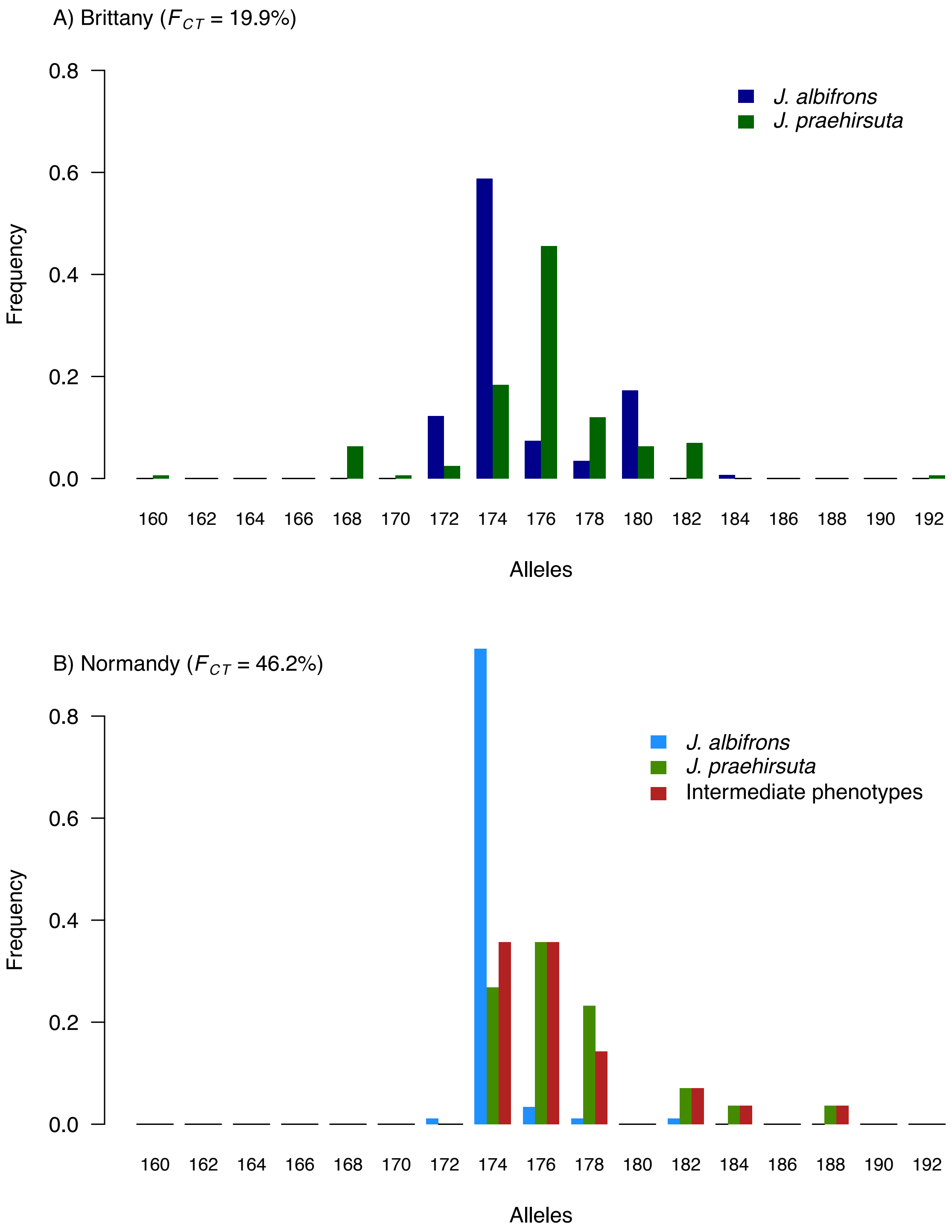
A) Brittany (F_{CT} = 19.9%)
J. albifrons
J. praehirsuta
Frequency
0.8
0.6
0.4
0.2
0.0
160 162 164 166 168 170 172 174 176 178 180 182 184 186 188 190 192
Alleles
B) Normandy (F_{CT} = 46.2%)
J. albifrons
J. praehirsuta
Intermediate phenotypes
Frequency
0.8
0.6
0.4
0.2
0.0
160 162 164 166 168 170 172 174 176 178 180 182 184 186 188 190 192
Alleles

Figure S5

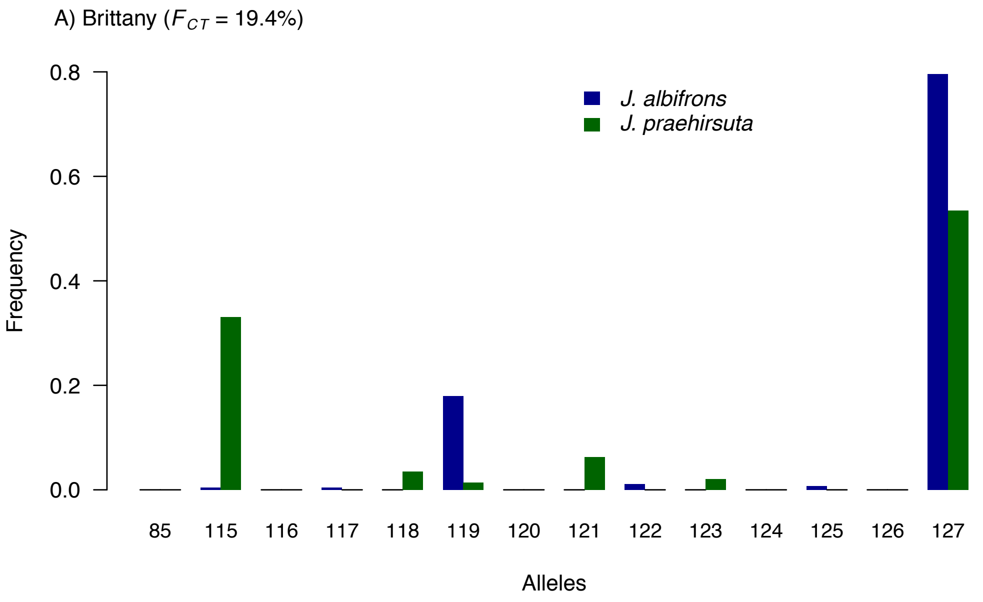
A) Brittany (F_{CT} = 19.4%)
J. albifrons
J. praehirsuta
Frequency
0.0
0.2
0.4
0.6
0.8
85
115
116
117
118
119
120
121
122
123
124
125
126
127
Alleles

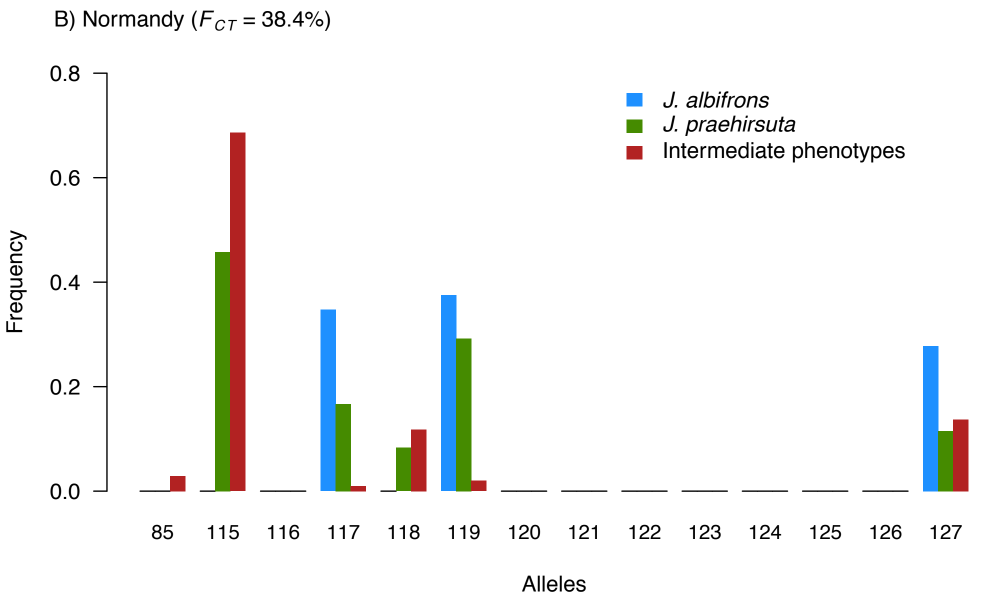
B) Normandy (F_{CT} = 38.4%)
J. albifrons
J. praehirsuta
Intermediate phenotypes
Frequency
0.0
0.2
0.4
0.6
0.8
85
115
116
117
118
119
120
121
122
123
124
125
126
127
Alleles

Figure S6

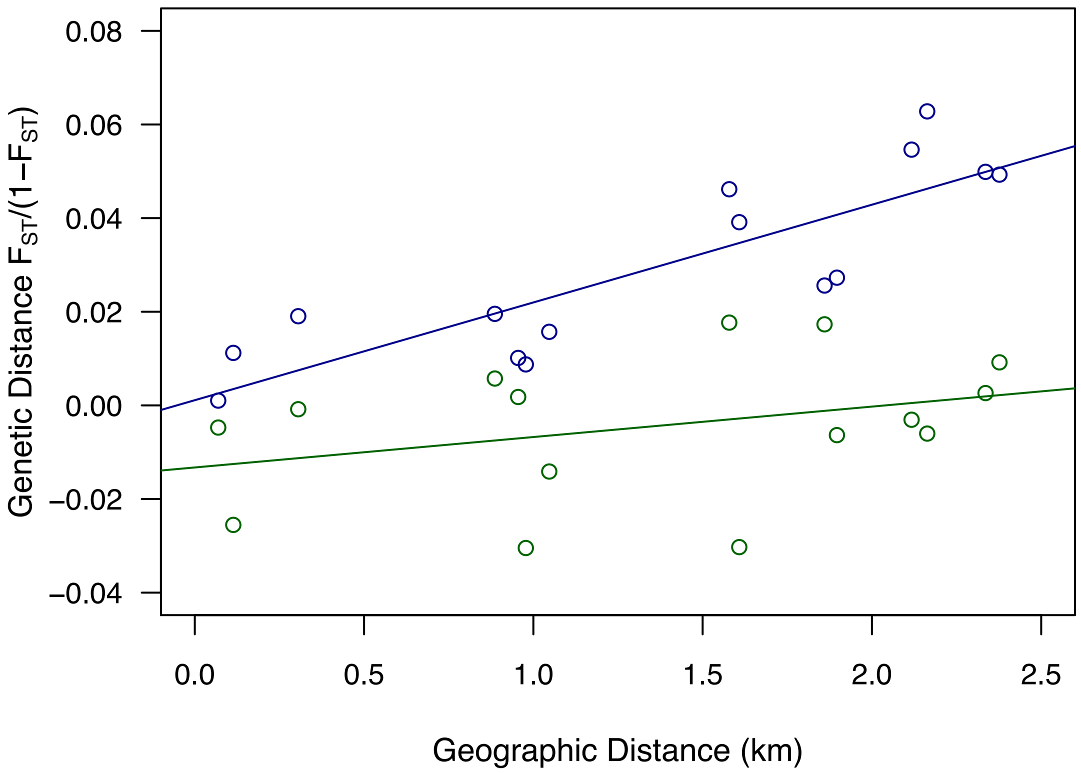

0.08
0.06
0.04
0.02
0.00
−0.02
−0.04
Genetic Distance $F_{ST}/(1-F_{ST})$
0.0
0.5
1.0
1.5
2.0
2.5
Geographic Distance (km)
